# A Working-Class View of Our Hospitals

**Published:** 1888-01-28

**Authors:** A. W. Webster

**Affiliations:** Chairman of Workmen's Governors, Sunderland Infirmary


### A Working-Class View of Our Hospitals.

By A. W. Webster, Chairman of Workmen's Governors, Sunderland Infirmary. (*Continued from p.* 288.)

The scheme propounded in my last article will probably be found most practicable for towns not exceeding medium size. Our great cities, such as London, Liverpool, Glasgow, etc., have so many charities equally worthy of support, that it would be manifestly unjust to attempt to direct the workmen's subscriptions to any one particular institution or class of institutions. My position as a workman has not given me the opportunity of studying the elaborate systems of organisation and representation, so adroitly brought into use for the benefit of political as well as charitable institutions, in most of our large centres of population, yet I may presume to make a few suggestions, based principally on local experience and observation.

It seems to me a possible thing in every large town except London for the committees of all the charities to meet in council and formulate a plan or plans by which every one of the institutions they represent might be permanently benefited. Let them start with the supposition (which I think will soon turn to fact) that the great majority of the workpeople are able and willing to support the charities from which they benefit so much. The town might be mapped out, certain works and localities being allotted to separate deputations, who would make it their duty to bring the penny per week system before the workmen employed within their respective areas (some attention being given to the hints in my last article).

A standard sum could be fixed to entitle to representation, and a central board of workmen's representatives might be formed, from which a fixed number of members of committee for each charity might be elected annually. The whole of the workmen's subscriptions might be centralized, and this workmen's board of representatives should have full power to allot the proportion to each charity. This scheme appears to me to be based on sound principles, and only requires the united action of the committees of charitable institutions in any one town to give it a start. Surely the grand army of philanthropists who spend their time and money year after year in the glorious work of ministering to the needy, will be able to bury their petty jealousies and rivalries for a season, and join hand in hand in initiating a scheme likely to revolutionise the financial position of all hospitals and kindred institutions. The building up of such a scheme, to approach anything like perfection, would of necessity be a gradual process. Probably no better method could be adopted in large towns than that of appointing a suitably paid secretary and collector, who could not only follow up the work of the special deputations chosen to launch the scheme in its earlier stages, but who would devote his whole time to maturing and extending it, and to the systematic collection of all monies contributed at the different workshops, etc.

When I turn my attention to London, that mighty metropolis of the world, I seem to face an unfathomable sea of difficulties on the one hand, and an endless sphere of possibilities on the other. Here we find wealth and influence in the closest proximity to penury and want; the greatest need linked to the greatest power to help; an immense heterogeneous mass of humanity, out of which almost anything within the powers of man may be evolved.

The hospitals of London have certainly been largely maintained by the aristocracy; yet what a miserable mite has been contributed in proportion to the gigantic wealth cased up within that few square miles of land, almost worth its weight in gold. But I am reminded that my editor has only commissioned me to deal with methods of raising income among the comparatively poor, and I must again drop suddenly from the munificence of the wealthy few to try and teach the doctrine that the workmen's pence may be more valuable than the rich men's thousands. Competent authorities inform me, and hospital statistics prove, that the workpeople of London contribute proportionately less to the support of their charities, and occupy them more, than those of any other part of the country. My somewhat limited knowledge of south-country workpeople may hardly entitle me to speak with authority, yet my occasional visits to London and my frequent intercourse with London workmen here, entirely fail to reveal anything within themselves to account for their non-support of such institutions. If I find the careful, saving disposition developed in the northern workmen, which enables some few of them to make better provision for times of sickness, I also find the free and open hand of the southerner, who would disdain to refuse his mite to hospital support if it were properly asked. The temptations constantly assailing the London workmen to spend their surplus cash are immensely more numerous than are to be found in the provinces; hence we find a difference of disposition, but that difference tends to prove that London workmen will yet support their hospitals more liberally than any others. Although it may be considered by some a practical impossibility to carry out in London the scheme suggested for other large towns, I have an impression that the careful guidance of a master mind or two could overcome all obstacles. London has its well-defined parliamentary boundaries, its municipal wards, and its parishes; it can be divided into twenty or thirty sections almost as easily as Liverpool or Glasgow. Each section might have its own secretary and collector, and its council of workmen's representatives, from which a few members of committee to each charity within its boundary lines could be elected annually.

The secretaries and, say, the chairmen and vice-chairmen of the workmen's councils in the twenty or thirty sections could then form a central council, which should have power to apportion the workmen's subscriptions to the various charities according to their claims. A scheme such as this needs an enthusiastic and persevering leader. Who will be its champion? Surely mighty London can produce him.

The Editor of The Hospital says London workpeople should contribute £80,000 or £90,000 per annum to their hospitals. I am inclined to predict that, with well-directed effort, he may yet have his heart gladdened by seeing that amount doubled. The central body, already partially clothed with the necessary authority, is in existence. Can the Hospitals Association not launch and man the scheme in its initiatory stages? Let their motto be one million pennies per week from the London workmen, and the Editor of The Hospital and a host of other editors will be found eager to assist in such a crusade.

I have advocated a centralised fund for London, notwithstanding the twenty or thirty sections into which I would recommend its division. I do this because of the concentration of particular classes in particular localities, and taking it altogether it will be found the most workable scheme, and the one most likely to give general satisfaction either in London or any other very large town. London hospitals have almost a world-wide claim for support, as they are more liable than any others to receive patients living at great distances beyond the Parliamentary boundaries of the City. This fact ought to extend the field for soliciting workmen's subscriptions, and should ever this or any similar scheme be adopted, I need only again assert that London, even with all its difficulties, presents one of the finest gathering grounds to be found in the world.

Begin the scheme, if you like, in the largest and most important workshops, but never think of stopping there. As soon as your Workmen's Charity Council becomes an accomplished fact, you will have secured the services of a sympathetic band of missionaries who will voluntarily assist the secretaries and hospital managers in extending the workpeople's subscriptions till hardly a draper's or milliner's shop in which a dozen workpeople are employed will remain outside the scheme. *(To be continued.)*

